# An Analysis of 1256 Cases of Sporadic Ruptured Cerebral Aneurysm in a Single Chinese Institution

**DOI:** 10.1371/journal.pone.0085668

**Published:** 2014-01-15

**Authors:** Lin Zhao, Lihong Zhang, Xiaolin Zhang, Zhenzhong Li, Linwei Tian, Yi-Xiang J. Wang

**Affiliations:** 1 Department of Neurosurgery, The Second Hospital of Hebei Medical University, Shijiazhuang, Hebei Province, China; 2 Department of Neurology, The Second Hospital of Hebei Medical University, Shijiazhuang, Hebei Province, China; 3 Department of Epidemiology and Statistics, School of Public Health, Hebei Medical University, Shijiazhuang, Hebei Province, China; 4 The Jockey Club School of Public Health and Primary Care, The Chinese University of Hong Kong, Hong Kong, China; 5 Department of Imaging and Interventional Radiology, Prince of Wales Hospital, The Chinese University of Hong Kong, Hong Kong, China; University Medical Center (UMC) Utrecht, Netherlands

## Abstract

**Background:**

To review the epidemiology of sporadic ruptured cerebral aneurysm.

**Methods:**

This is a retrospective study of consecutive 1256 Chinese patients between January 2006 and January 2013, who were admitted to the Second Hospital of Hebei Medical University, China, for spontaneous subarachnoid hemorrhage due to a rupture of cerebral artery aneurysm. In 288 males and 478 females, the size of aneurysms was measured by a neuroradiologist on DSA. In 123 males and 184 females, the size of the ruptured aneurysms was not measured. The remaining patients, with 61 males and 122 females, had multiple aneurysms, and the medical record could not reliably determine the specific aneurysm responsible for the rupture.

**Results:**

In total there were 784 females and 472 males with a female/male ratio of 1.66. The female/male ratio was down to 0.50 for patients younger than 35 yrs. For both males and females, aneurysm rupture was most common during the age of 50–59 yrs. Ruptured aneurysms were mostly of 2 mm–5 mm in size (47.1%), followed by 5 mm–10 mm (39.7%). Ruptured single cerebral aneurysm occurred in anterior circulation in 95.0% of the cases, with 5.0% occurred in posterior circulation. Ruptured aneurysm most commonly occurred at posterior communicating artery (34.9%) and anterior communicating artery (29.5%). 183 cases (14.6%) had multiple aneurysms.

**Conclusions:**

With younger patients, there is a male predominance in our series. Ninety percent of patients have ruptured aneurysms less than 10 mm in size.

## Introduction

Ruptured cerebral aneurysm is the most common cause of subarachnoid hemorrhage (SAH), causing significant morbidity and mortality. The incidence of SAH in western populations is about 9 to 15 per 100,000 persons per year [1]–[3]. The epidemiology of cerebral aneurysm in western populations is well reported in the literature [4]–[7]. Some studies attempted to determine whether there is a critical size at which an aneurysm is likely to rupture and thus warrant treatment [8]. It has also been reported that there is a higher incidence of rupture of cerebral aneurysms in patients in Japan [9]. An annual risk of cerebral aneurysm rupture of 2.7% has been reported in Japan, and this is relatively high compared to results from Europe and North America. Rinkel *et al* reported an annual risk of rupture of 1.9% in Western populations [4], [9].

Unlike Caucasian and Japanese populations [1]–[12], few studies describing the epidemiology of cerebral aneurysm in the Chinese population have been published [13]–[15]. The purpose of this study is to review the epidemiology of sporadic ruptured cerebral aneurysm, in terms of size, location, the prevalence of multiple cerebral aneurysms, and cerebral aneurysm's gender difference in Chinese population. Reliable knowledge about the risks of cerebral aneurysm will help in planning, screening and prevention strategies and in predicting the prognosis of individual patients.

## Patients and Methods

This is a retrospective study of consecutive 1256 Chinese patients with ruptured cerebral aneurysm between January 2006 and January 2013, who were admitted to the Second Hospital of Hebei Medical University which is a tertiary referral center of neurological diseases in Northern China, for spontaneous SAH due to a rupture of cerebral artery aneurysm. The data used in this study was retreated from the medical records of the hospital. The institutional review board of the Second Hospital of Hebei Medical University approved this retrospective analysis, and informed consent was waived. The patients included 472 males and 784 females, with mean age of 53.85 yrs (SD: 10.64 yrs, range: 14–88 yr). SAH was initially diagnosed by brain computed tomography, and digital subtraction angiography (DSA) was followed and SAH was confirmed to be due to cerebral aneurysm. In 288 males and 478 females (group 1), the size of the aneurysms was measured by a neuroradiologist at the time of diagnosis, and we used the measurement the neuroradiologist reported. The measurement was done on DSA with the largest diameter measured through the long axis of the aneurysm [6], [15]. In 123 males and 184 females, the size of the ruptured aneurysm was not measured though the location of the ruptured intracranial aneurysms was recorded (group 2). The remaining patients, with 61 males and 122 females, had multiple aneurysms present (group 3), and our medical record could not reliably determine the specific aneurysm responsible for the rupture.

All statistical analyses were performed with SPSS 14.0 for Windows (SPSS, Inc., Chicago, IL). Group comparisons were performed with Student's t-test for normally distributed continuous variables or Mann-Whitney U test for other continuous variables. Contingency tables were analysed with Fisher's exact test for dichotomized variables or χ^2^ statistics. *P*<0.05 was considered statistically significant.

## Results

### 1. Gender characteristics in ruptured cerebral aneurysm patients

The total 1256 patients (inclusive of groups 1–3) had 784 females and 472 males, with a female/male ratio of 1.66, indicating overall females had a higher cerebral aneurysm rupture incidence than males (*p*<0.0001, Table 1). The female/male ratio was 0.72 for patients younger than 40 yrs, and down to 0.50 for patients younger than 35 yrs (*p*<0.05), indicating in younger patients males had a higher cerebral aneurysm rupture incidence than females (Table 1).

**Table 1:** Female male ratio in ruptured cerebral aneurysm patients.

	Female	Male	Female/male ratio	*p*
Total patients (n = 1256)	784	472	1.66	<0.0001
Patients ≤39 yrs (n = 105)	44	61	0.72	0.097
Patients ≤34 yrs (n = 45)	15	30	0.50	0.025

### 2. Age characteristics in ruptured cerebral aneurysm patients

**Figure 1:**
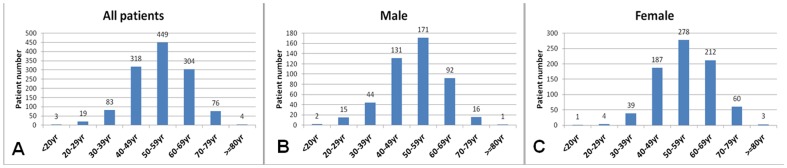
Age characteristics in the ruptured cerebral aneurysm patients. A: age distribution for all 1256 patients; B: age distribution for 472 male patients; C: age distribution for 784 female patients.

The age distribution of the total 1256 patients (inclusive of group 1–3) is shown in Figure 1. For both males and females, aneurysm rupture was most common during the age of 50–59 yrs. There were subjects who suffered ruptured aneurysm before the age of 30 yrs (n = 19) and before the age of 20 yrs (n = 3). The mean age of male patients was significantly lower than that of female patients (51.6±11.0 yrs vs 55.2±10.2 yrs, *p*<0.001).

### 3. Size characteristics in ruptured single cerebral aneurysm patients

The size distribution of ruptured single cerebral aneurysm patients (group 1, total = 766) was: >25 mm: 4 aneurysms (0.52%); >20 mm: 5 aneurysms (0.65%); >10 mm: 63 aneurysms (8.22%); >5 mm: 304 aneurysms (39.69%); >2 mm: 361 aneurysms (47.13%); and ≤2 mm: 29 aneurysms (3.79%). The mean ruptured aneurysm size was 6.01 mm, with 6.17 mm (median 5.45 mm) for males, and 5.91 mm (median 5.00 mm) for females. There was no significant difference in ruptured aneurysm size between males and females (Table 2, *p*>0.05). Ruptured aneurysms were most likely in the region of 2 mm–5 mm (47.13%), followed by 5 mm–10 mm (39.69%). Twenty nine patients (3.79%) had ruptured cerebral aneurysm sized less than 2 mm. The trend that males had a larger aneurysm size than females was seen in younger subjects (Table 2). In female subjects, a trend of smaller ruptured aneurysm in younger subjects was seen, while this trend was not observed in males (Table 2).

**Table 2:** Size of ruptured single cerebral aneurysm for male and female patients at different age groups.

	Mean size (median, SD)		Mean size (median, SD)	*p*
Male total (n = 288)	6.17 (5.45; ±3.87) mm	Female total (n = 478)	5.91 (5.00; ±3.53) mm	>0.05
Male ≤39 yrs (n = 42)	5.83 (4.95;±3.64) mm	Female≤39 yrs (n = 31)	4.48(4.00; ±2.13) mm	0.05
Male ≤34 yrs (n = 22)	6.73 (5.65; ±4.07) mm	Female≤34 yrs (n = 11)	3.48 (3.00; ±1.53) mm	<0.05
n = 5 males	Aneurysm>20 mm	n = 4 females	Aneurysm>20 mm	

*p* : male vs female comparison.

### 4. Location of ruptured single cerebral aneurysms

The analysis of location of ruptured cerebral aneurysms included group 1 and group 2 patients (n = 1073). Ruptured single cerebral aneurysm occurred in anterior circulation in 95.0% of the cases, while 5.0% occurred in posterior circulation (Table 3). Ruptured aneurysms most commonly occurred in posterior communicating artery (PcoA, 34.86%) and anterior communicating artery (AcoA, 29.45%). There were more cases of AcoA aneurysm rupture before the age of 50 than PcoA aneurysm rupture (149∶80), while there were more cases of PcoA aneurysm rupture after the age of 50 than AcoA aneurysm rupture (294∶167, Table 3).

**Table 3:** Location of ruptured single cerebral aneurysms.

Anterior circulation	ICA	MCA	ACA	AcoA	PcoA	AChA	PA	CMA	Sub-total
<40	12	14	2	43	13	0	3	0	87
40–49	34	48	12	106	67	1	1	2	271
50–59	41	58	7	98	148	1	2	0	355
60–69	35	27	7	58	113	1	0	0	241
> = 70	10	9	2	11	33	0	0	0	65
Sub-total	132	156	30	316	374	3	6	2	1019
Posterior circulation	VA	BA	PCA	PICA	SCA	Sub-total			
Sub-total	12	19	14	7	2	54			

ICA: internal carotid artery (including any aneurysm located in the intracranial portion of ICA), MCA: middle cerebral artery, ACA: anterior cerebral artery, AcoA: anterior communicating artery, PcoA: posterior communicating artery, AChA: anterior choroidal artery, PA: pericallosal artery, CMA: callosomarginal artery, VA: vertebral artery, BA: basilar artery, PCA: posterior cerebral artery, PICA: posterior inferior cerebellar artery, SCA: superior cerebellar artery.

The size of ruptured single cerebral aneurysm at different arteries is shown in Table 4. The aneurysms at anterior choroidal artery and pericallosal artery tended to have a smaller size, followed by aneurysms at anterior cerebral artery (Table 4).

**Table 4:** Size of ruptured single intracranial aneurysm at different arteries.

Anterior circulation	ICA	MCA	ACA	AcoA	PcoA	AChA	PA	CMA
Mean size (±SD) mm	7.51 (±5.95)	5.39 (±3.09)	4.50 (±2.36)	5.18 (±2.77)	6.53 (±3.28)	3.50	3.30 (±1.18)	6.60
Posterior circulation	VA	BA	PCA	PICA	SCA			
Mean size (±SD)	7.20 (±1.74)	6.31 (±3.00)	6.40 (±3.99)	6.73 (±4.12)	7.25			

Abbreviations see table 3.

### 5. Characteristics of multiple cerebral aneurysms

In the total 1256 patients, 183 cases (14.57%) had multiple aneurysms, with 61 (61/472, 12.92%) in male patients and 122 (122/784, 15.56%) in female patients. There was no difference of multiple aneurysms prevalence in males and females (*p* = 0.2). In these 183 patients, 159 patients (86.89%) had two aneurysms; the remaining 24 patients (13.11%) had more than two aneurysms. The mean age of single aneurysm patients (mean age = 53.50±10.72) was slightly younger than those with multiple aneurysms (mean age = 55.92±9.87, *p* = 0.003). The site information of multiple aneurysms is shown in Table 5. Multiple aneurysms occurred in anterior circulation in 91.8% cases, and in posterior circulation in 8.2% cases. Multiple aneurysm most commonly occurred in PcoA (38.62%), followed by internal carotid artery (23.02%) and medial cerebral artery (13.81%).

**Table 5:** Location of multiple aneurysms.

Anterior circulation	ICA	MCA	ACA	AcoA	PcoA	AChA	PA	CMA	Sub-total
Males	29	22	7	18	36	0	1	0	113
Females	61	32	12	21	115	1	3	1	246
Sub-total	90	54	19	39	151	1	4	1	359
Posterior circulation	VA	BA	PCA	PICA	SCA				Sub-total
Males	4	5	1	0	4				14
Females	1	10	4	1	2				18
Sub-total	5	15	5	1	6				32
Mirrored aneurysms	ICA	MCA	ACA	PcoA	VA	PCA	SCA		Sub-total
Males	4	4	0	8	0	0	1		17
Females	3	1	1	36	0	1	0		42
Sub-total	7	5	1	44	0	1	1		59

Abbreviations see table 3.

Of the multiple aneurysm cases, 59 cases had mirrored aneurysms (32.24% out of the 183 cases with multiple aneurysms, and 4.7% out of the total 1256 cases), where aneurysms distributed both on the right side and left side in a mirrored manner [16]–[18]. With the cases of mirrored aneurysms, 44 cases (74.6%) occurred in PcoA (Table 5). There was no significant difference of incidence rate for mirrored aneurysm between males and females (*p*>0.05).

## Discussion

To our knowledge, this is largest retrospective analysis on the epidemiology of ruptured cerebral aneurysms in the Chinese population with SAH. One comparable study in Chinese population is the Hong Kong study of 267 Chinese patients with SAH from ruptured cerebral aneurysms [15]. The patients in our series ranged in age from 14 to 88 yrs with a mean age of 53.9 yrs, which is slightly younger than the mean age of 59 yrs reported by the Hong Kong study [15], while older than the mean age of ruptured cerebral aneurysms for Caucasian patients [19], [20]. Weir et al reported that in a database of 945 patients, the average age of patients with ruptured aneurysms was 46 yrs [19]. In Aarhus et al's study, the median patient age was 50.9 yrs [20]. The same as the Hong Kong study [15], males presented with ruptured cerebral aneurysms at a younger mean age (51.6 yrs) than females (55.2 yrs). This observation has also been reported in Western literature. Aarhus et al [20] reported male patients were younger than female patients [48.2 yrs vs. 53.8 yrs]. A female predominance of patients with ruptured cerebral aneurysms has been reported in studies from the West [8; female to male ratio: 2.86∶1], Japan [21; female to male ratio: 1.46∶1], and Taiwan [14, female to male ratio 1.47∶1.]. With the total 1256 patients in our series, the female to male ratio was 1.7∶1. However, our results showed with younger patients (≤39 yrs), there was a male predominance, and the trend was more apparent when even younger patients (≤34 yrs) are considered (Table 1). The Hong Kong study demonstrated a trend of larger ruptured aneurysms in men (mean size, 6.3 mm) than in women (mean size, 5.6 mm), however, statistical significance was not achieved. This study demonstrated that males also had a slight larger aneurysm size (mean size, 6.17 mm) than females (mean size, 5.91 mm), again statistical significance was not achieved. However, males had a larger aneurysm size than females in younger subjects with statistical significance (*p*<0.05 for subjects less than 35 year old, Table 2).

Many series, including this study, demonstrated that the majority of ruptured aneurysms are less than 10 mm in diameter. In this series, 90.6% (694/766) of the patients had ruptured aneurysms sized ≤10 mm. 50.9% (390/766) of the patients in our series had ruptured aneurysms sized ≤5 mm. Previous study in the Chinese population demonstrated a proportion of 64% had aneurysms of size 5 mm or less [15]. This is different from the results in western and Japanese populations, where it was reported a lower proportion of ruptured cerebral aneurysms had a size of 5 mm or less [6], [21]–[25]. Kassel and Torner analyzed 1092 patients with SAH and reported that 71% of the aneurysms were less than 10 mm in diameter and 13% were less than 5 mm in diameter, respectively [25]. For female subjects, trend of smaller ruptured aneurysms in younger subjects is seen in this study (Table 2).

While some studies classified PcoA as part of the posterior circulation [26], the PcoA connects the posterior and anterior cerebral circulations and almost all of the aneurysms that affect it arise at the anterior circulation end and such is usually considered part of the anterior circulation [27]. In our study, we classified PcoA as part of the anterior circulation. A high proportion of ruptured aneurysms located in the PcoA and AcoA, which is similar to the pattern reported in western and Japanese populations [6], [19], [21]. Such findings are also consistent with previous literatures from Hong Kong and Taiwan [13]–[15]. Our data showed there were more cases of AcoA aneurysm rupture before the age of 50 than PcoA aneurysm rupture, while there were more cases of PcoA aneurysm rupture after the age of 50 than AcoA aneurysm rupture (Table 3).

The prevalence of multiple aneurysms in our series was 14.57%. This is broadly similar to the previous report of 17% in Hong Kong population and 15% in Japanese population [15], [28], whilst some literature describing western populations reported this figure to be 30–40% [17], [29]–[31]. Some reports listed the risk factors of multiple aneurysms, including smoking, hypertension, and family history of cerebrovascular diseases [30], [31]. In the Hong Kong series [15], there was no significant difference in the incidence of multiple aneurysms between men and women, which is contrary to the general finding that female gender is a risk factor for multiple aneurysms [29], [31]. The current study also showed more females had multiple aneurysms than males (Table 5), however this was due to overall females had a higher incidence of ruptured aneurysms, and there was no statistical difference in incidence of multiple aneurysms between men and women in this study. ‘Mirror-like’ aneurysm, which are located bilaterally on corresponding arteries, has been reported to constitute less than 5% of overall aneurysm [17], [18]. In this series of ruptured cerebral aneurysms, 4.7% (59/1256) had mirrored aneurysms and dominantly occurred in PcoA (44/59). This result is different from Meissner et al's report that the most common distribution for mirror aneurysms was the middle cerebral artery followed by noncavernous internal carotid artery [16]. It has been reported that the presence of a mirror aneurysm is not an independent predictor of future SAHs [16]. The prevalence of giant aneurysms (sized >25 mm) in our series (0.52%) was slightly lower than previous reports on Hong Kong Chinese (1%) and the Japanese population (1%) [15], [21], and substantially less than the figure of approximately 4% published in literature from the West [6].

There are many limitations with our study. This is a retrospective analysis of the medical record of a single hospital. In 39.01% of patients the size of rupture aneurysms was not recorded. Also in multiple aneurysms the specific aneurysm for the rupture could not be asserted. Screening for asymptomatic cerebral aneurysms is not routinely undertaken in China. Our study simply examined ruptured aneurysms in a population with an unknown number of unruptured aneurysms. Furthermore, we do not know how many patients with ruptured aneurysms did not seek medical attention. Hence, the risk of rupture of those unruptured aneurysms cannot be extrapolated from the data of patients with ruptured aneurysms. However, despite these limitations, our data provided interesting and important data of the epidemiology of ruptured cerebral aneurysms in Chinese population.

## Conclusions

This study indicates in younger subjects males has a higher cerebral aneurysm rupture incidence than females. Aneurysm rupture is most common during the age of 50–59 yr, and the mean age of male patients is younger than that of female patients. A high proportion of the ruptured aneurysms in our series have a size less than 5 mm. Ruptured aneurysms most commonly occur in AcoA and PcoA. About fifteen percent of patients in our series have multiple aneurysms.
